# Cyber Bullying Prevention: Intervention in Taiwan

**DOI:** 10.1371/journal.pone.0064031

**Published:** 2013-05-28

**Authors:** Ming-Shinn Lee, Wu Zi-Pei, Leif Svanström, Koustuv Dalal

**Affiliations:** 1 Department of Curriculum Design and Human Potentials Development, National Dong Hwa University, Hualien, Taiwan; 2 Department of Public Health Science, Karolinska Institutet, Stockholm, Sweden; 3 Centre for Injury Prevention and Safety Promotion (CIPSP), School of Health and Medical Sciences, Örebro University, Örebro, Sweden; The University of Queensland, Australia

## Abstract

**Background:**

This study aimed to explore the effectiveness of the cyber bullying prevention WebQuest course implementation.

**Methodology/Findings:**

The study adopted the quasi-experimental design with two classes made up of a total of 61 junior high school students of seventh grade. The study subjects comprised of 30 students from the experimental group and 31 students from the control group. The experimental group received eight sessions (total 360 minutes) of the teaching intervention for four consecutive weeks, while the control group did not engage in any related courses. The self-compiled questionnaire for the student’s knowledge, attitudes, and intentions toward cyber bullying prevention was adopted. Data were analysed through generalized estimating equations to understand the immediate results on the student’s knowledge, attitudes, and intentions after the intervention. The results show that the WebQuest course immediately and effectively enhanced the knowledge of cyber bullying, reduced the intentions, and retained the effects after the learning. But it produced no significant impact on the attitude toward cyber bullying.

**Conclusions/Significance:**

The intervention through this pilot study was effective and positive for cyber bulling prevention. It was with small number of students. Therefore, studies with large number of students and long experimental times, in different areas and countries are warranted.

## Introduction

Bullying is one of the most significant school health problems among children and adolescents. Literature indicated that bullying is a “systematic abuse of power” in an asymmetric power relation between the victim and the bully [1]. The victim belongs to a physically or psychologically weaker minority group than the aggressor (bully). Bullying is a set of repeated actions of bully against the bullied (victim) [2].

Depending on the means and ways, bullying can generally be divided into six types: verbal-bullying, physical-bullying, sexual-bullying, social-bullying, defensive-bullying, and cyber bullying. Cyber bullying takes place in cyber space. Cyber bullying is arising from the popularization of the computer network and communication technology in recent years is also known as electronic bullying, SMS bullying, digital bullying, or online bullying.

Bullying has several social, emotional, mental health and academic effects [3]–[10]. It results with depression, anxiety (individual and social), aggression, hyperactivity, academic discomfort and conduct problems at school [3], [9], [10]. However, cyber victims demonstrated a higher level of depression than other forms of bullying [11].

Cyber bullying has been increasing rapidly for the last few decades with the intense use of internet, sms, mms and other electronic media. It has been emerged as a major concern for the teachers, academicians and school authorities. Hence, the growing prevalence of cyber-bullying demands adequate research for prevention including mainly teachers and academicians. Teaching young students the correct knowledge and attitude toward cyber bullying control has become an important issue.

WebQuest is a theme-based teaching activity that has been widely accepted by scholars and teachers [12]. This teaching strategy aims to assist students engaging in exploratory learning activities through network resources. Teachers design the targets of tasks to help learners concentrate more effectively on information use rather than the search for information and assist them to engage in higher levels of analysis, integration, and assessment-based thinking activities.

Cyber bullying media varies with the different habits of the technology-use in different regions, including use of email, blogs, websites, chat rooms, mobile phones, instant messaging, web pages, text messages, online voting websites, and online games. Cyber bullying comes in quite diverse forms such as online debates of mutual accusations, harassment, slander, imitation, disclosure, fraud, exclusion, and network tracking [13]. In Taiwan, cyber bullying is one of the major school health problems [14]. WebQuest is a teaching activity that assists students, engaging in online (cyber) exploratory-oriented teaching activities. Cyber bullying problems encompass within cyber world. In the current study, an effort was mead in Taiwan school to use the WebQuest course, designed to determine if the course does indeed contribute to the student’s correct knowledge and attitude toward cyber bullying, as well as their intentions to reduce cyber bullying.

## Methods

### The Concept and Framework of WebQuest

WebQuest is a set of student-centered and exploration-oriented learning activities presented in a webpage layout. Based mainly on social constructivism, scaffolding theory, and collaborative learning theory WebQuest has six components introduction, task, process, resources, assessments, and conclusions. WebQuest has been developed to cover all educational themes. It has been rapidly popularizing and appling in various types of teaching [15]. In Taiwan, WebQuest has been used in different fields covering language, science, social studies, hygiene and health, art and music, business and economy, etc. [16]–[21].

### Implementation Period and Study Subjects

This research was implemented during the second semester of 2010 among seventh grader. Students of two classes of seventh graders in same were the study subjects. One class of 30 students was experimental group, adopted with the WebQuest teaching design. Another class of 31 students was control group. The teaching time comprised of 8 lessons (each lesson was 45 minutes long).

### Research Tools

In this study, the self-compiled “questionnaire for the youth’s categories of the family social-economic status, internet usage, knowledge, attitudes, intentions toward cyber bullying prevention” was adopted as the survey tool. The questions of internet usage consisted of availability of home computer, availability of internet at home, experiences of internet usage, the average hours of internet usage, the main purposes of internet usage. This questionnaire was established after undergoing expert validity and content validity ICVI and the pre-test. The Cronbach α coefficient for internal consistency was 0.79, the attitude scale was 0.88, and the intentions survey was 0.93, indicating considerable internal consistency.

The various research tools employed in the current study are described as follows:

#### i) The “knowledge test” of the youth’s cyber bullying prevention

The test content was designed for young people’s understanding. Targeting the questionnaire contents and based on the information ability indicators in sequences for middle school seventh graders, suitable questions were selected to make textual changes in consultation with bullying scale [22] and “Questionnaire of Information Ethics for Senior Elementary School Students” [23]. The questionnaire content consists of 17 questions including “cyber bullying knowledge,” “network etiquette,” “Internet security,” “Internet law,” etc. Each question lists four options, only one of which is the correct answer. For every correctly answered question, five points are given; no point is awarded for a wrong answer. Therefore, the higher the test score, the higher was the student’s knowledge level and vice-versa.

#### ii) The “attitude scale” of the youth’s cyber bullying prevention

In this questionnaire, the attitude toward cyber bullying was to investigate the student’s views during computer network access. In consultation with “scale of perception and response toward cyber bullying” [24] and “questionnaire survey of elementary school children’s network literacy and network ethics” [25] four scales totaling of 22 questions were developed for the current study: cyber bullying, network etiquette, network security, and network law. The 5-point Likert Scale was adopted for answering options. The answer options included: “strongly disagree (1)” to “strongly agree (5)”. The higher the score, the more positive was the student’s attitude toward cyber bullying and vice versa.

#### iii) The “intentions survey” of the youth’s cyber bullying prevention

The cyber bullying intentions survey aspect targeted a forecast of cyber bullying behaviors in the near future, to explore whether the youth had ever come across the idea of engaging in cyber bullying. Researchers deemed bullying behaviors as a type of attack behavior classified into two: reactive attacks and proactive attacks [26], [27]. The current study content includes proactive attacks and reactive attacks, a total of 8 questions [28]. Each question lists five Likert scale options. From the five options of “entirely inconsistent,” “mostly inconsistent,” “somewhat consistent,” “mostly consistent,” and “entirely consistent,” the respondent selected the answers that best described their situations. As to the degree of perception from low to high, scored from 1 to 5, the scores corresponding to the answers selected by the respondent were added. The higher the score, the more obvious was the respondent’s characterized cyber bullying intentions. The lower the score, the less obvious was the respondent’s characterized cyber bullying intentions.

### The WebQuest Course Content

In the study, the WebQuest course content covers six parts: introduction, tasks, process, resources, assessments, and conclusions. The contents are presented in Table 1.

**Table 1:** Contents of the cyber bullying WebQuest course.

1.Introduction	The researcher guides the students to think about their daily computer network use to engage in with this learning activity.
2. Tasks	The students are divided into teams to complete the four tasks assigned in this study through the collaborative learning model
	Task 1. Network pickets: As pickets, the usual use of the computer network is checked, and network etiquette is checked through group discussion of the cases.
	Task 2. The news chase: As newscasters, the roles of cyber bullying events are analyzed, the ways to respond to cyber bullying are proposed, response and protection strategies are put forward.
	Task 3. Network law enforcement: To understand legal-violations-related issues and to engage in the “question-and-answer quiz” activity. With “network security” as the theme, the team members create propaganda posters.
3. Processes	To clearly describe the procedures and steps which students go through to complete the task.
4. Resources	To provide resources needed to complete the task. The resources include web pages, documents, databases, etc.
5.Assessments	To set up a set of assessment standards to enable students to understand the various assessment criteria for task results in advance.
6. Conclusions	To guide the students to reflect and think throughout the learning process and present feedbacks and reviews.

### Study Design

#### The quasi-experimental study design

In this study, the purposive sampling was adopted to select the seventh grader students. While the experimental group engaged in the 4-weeks WebQuest cyber bullying prevention course, the control group did not undergo any experimental processes. The nonequivalent group’s pre-test and post-test designs are depicted in Table 2. The students in the experimental and control group all underwent the pre-test. At the end of the course, the post-test was immediately conducted for the two groups of students. Two weeks after the end of the course, the two groups of students then underwent the follow-up test. The scores of the experimental group in the pre-test (O1), post-test (O2), and follow-up test (O3) and the scores of the control group in the pre-test (O4), post-test (O5), and follow-up test (O6) were all obtained from the “the questionnaire for the youth’s knowledge, attitudes, and intentions toward cyber bullying prevention.”

**Table 2:** Quasi-experimental nonequivalent group’s pre-test and post-test design.

	Pretest	Course Intervention	Post-test	Follow-uptest
Experimental Group	O_1_	X	O_2_	O_3_
Control Group	O_4_		O_5_	O_6_

### Statistical Analysis

The frequency distribution in percentages was adopted to describe the distribution of the basic information. The GEE model estimates the effect of intervention averaged over all the clusters. The generalized estimating equation (GEE) method is widely used to fit the time trend in repeated measurements due to its robustness to random missing and misspecification of the true correlation structure [29]. The variance of the estimated regression coefficient from an averaged model can be obtained using either model-based or robust estimators [30]. The compound symmetry pattern provides the correlations equal for any two measurements on the same subject. Both individual-level and cluster-level covariates can be incorporated in GEE. Therefore, GEE was adopted to analyze the differences among the pre-test, post-test, and follow-up test. α = .05 was used throughout the analysis.

To illustrate sample size estimation for a dichotomous longitudinal outcome, we considered estimating the sample size for the study in 2 groups measured at 3 time points. Due to lack of similar study, we set the effect sized at 0.3, α = 0.05, the power at 80% and get that the total sample size is 20. However, the original class size was 30 students from the experimental group and 31 students from the control group, totaling the size at 61 students. The IBM SPSS 19 version was used for the statistical analysis in this study.

### Ethical Issues

Ming-Shinn Lee and Wu Zhi-Pei had received the approval from National Dong Hwa University, Department of Curriculum Design & Human Potentials Development graduated student research review board. They had a formal meeting with the teacher, explained and showed all the curriculum, content and the protection strategy. Informed written consent was obtained from the teachers. Also to mention here that the Government of Taiwan (ROC) order, (DOH Document:1010064538) mentioned: “…Involving Social behavior sciences are included in the specification, the controversy still is large, and therefore not included in this human research law applies…”.

## Results

There were 27 boys (44%) and 34 girls (56%). The categories of the family status were: 49 students having parents (80.33); 35 students from families of low socioeconomic status (578%); 16 students from middle socioeconomic families (26%); 10 students from high socioeconomic families (16%). There were 18 students whose internet usage was less than one hour per day (30%), 31 students whose internet usage was one to three hours per day (51%), six students whose internet usage was four to six hours per day (10%), two students with seven to nine hours per day (3%) and four students with more than nine hours per day (7%). The basic background information for the experimental and the control groups were homogeneous, except that there were significant differences in the socioeconomic status among the two group members.

### WebQuest Course in the Knowledge Aspect for Cyber-bullying Prevention

As shown in Table 3, the generalized estimating equation analyses found that there was a significant difference in the pre-test scores between the experimental and the control groups. In other words, the knowledge of the experimental and the control groups were significantly different before the course and teaching. With controlling the scores of the pre-test, the post-test, the follow-up posttest, and the interaction between group and time, the mean difference in the score of knowledge between the experimental and the control groups was 8.58, which was significantly different (p = .028). With controlling pre-test, group, and the interaction of group and time, there was a significant difference in the score of knowledge for the post-test and the follow-up posttest. The scores for the post-test increased by 24.26 and for the follow-up posttest by 20.39. Therefore, the scores for the post-test and the retention after learning between the experimental and the control groups had significant differences. However, the interaction of group and time was not significantly different (p = .784). That was, the scores of knowledge between the experimental and the control groups did not differ as the time of tests differed.

**Table 3:** The generalized estimating equation analyses of the *knowledge aspect* of the cyber bullying prevention WebQuest course (n = 61).

Variable	Coefficient estimate	Standard error	Wald	*P* value
Score of the knowledge test				
Group	8.58	3.90	1.48	.028
Pretest	.62	.09	2.57	.000
Post-test T_1_	24.26	6.54	1.93	.000
Post-follow up test T_2_	20.39	6.94	2.41	.003
Group×Time	−.80	2.91	.52	.784

### WebQuest Course in the Attitude Aspect for Cyber-bullying Prevention

As shown in Table 4, the results of the generalized estimating equation analyses showed that the pretest scores between the two groups were significantly different; i.e. the attitudes of the two groups towards cyber-bulling were significantly different before course teaching. With controlling the scores of the pretest, the post-test, the post-posttest, and the interaction of group and time, the mean difference in the scores of the attitude scale between the experimental and the control groups was 2.87, which was not significantly different. With controlling pretest, group, the interaction of group and time, the scores of the attitude scale for the post-test and the post-posttest were not significantly different after the teaching of the WebQuest course for cyber-bulling. The score for the post-test increased by 13.20, which is not significantly different (p = .114); and the score for the follow-up posttest increased by 10.07, which is not significantly different (p = .229). The interaction of group and time was not significantly different (p = .844). In other words, the scores of the attitude scale between the experimental and the control groups did not differ as the test time differed.

**Table 4:** The generalized estimating equation analyses of the *attitude aspect* of the cyber bullying prevention WebQuest course (n = 61).

Variable	Coefficient estimate	Standard error	Wald	*P* value
Score of the attitude scale				
Group	2.87	2.97	.98	.334
Pretest	.86	.08	3.41	.000
Post-test T_1_	13.20	8.34	1.26	.114
Post-follow up test T_2_	10.07	8.38	1.10	.229
Group×time	.40	2.01	.45	.844

### WebQuest Course in the Intention Aspect for Cyber-bullying Prevention

As shown in Table 5, the results of the analyses on the cyber-bulling intention aspect of the WebQuest course for cyber-bulling prevention showed that the scores of the pretest for the two groups were significantly different, i.e. the cyber-bulling intentions of the two groups were significantly different before course teaching. With controlling the scores of the pretest, the post-test, the post-posttest, and the interaction of group and time, the mean difference in the intention score between the experimental and control groups was −2.49, which was not significant. With controlling pretest, group, the interaction of group and time, there was a significant difference for the post-test and the post-posttest. The score for the post-test decreased by 5.39, which was significant (p = .005); and the score for the follow-up posttest decreased by 5.81, which was significant (p = .002). Apparently, there was a significant difference between the experimental and the control groups at the post-test and after learning retention. The interaction of group and time was not significant (p = .962). In other words, the intention scores for the experimental and control groups were not affected by the test time.

**Table 5:** The generalized estimating equation analyses of the *intention aspect* of the cyber bullying prevention WebQuest course (n = 61).

Variable	Coefficient estimate	Standard error	Wald	*P* value
The intention score				
Group	−2.49	1.95	1.13	.201
Pretest	.63	.15	2.02	.000
Post-test T_1_	−5.39	1.93	1.67	.005
Post-follow up test T_2_	−5.81	1.89	1.75	.002
Group × time	−.05	1.09	.20	.962

## Discussion

Cyber bullying prevention program using WebQuest is successful. Cyber-bulling prevention WebQuest course can immediately enhance the knowledge and reduce the intention of cyber-bulling and is successful for retaining learning effects. To the best authors knowledge, first time an effort was made to utilize WebQuest course to educate school students for cyber-bulling prevention. However considering the small-scale study the current effort can be seen as a pilot study, warranting large-scale studies for further generalization.

The results have indicated that the course can immediately and effectively enhance the knowledge of cyber-bulling and has after learning retention effects. These findings are same as the results of the research by Hsing-Fang [31], but different from that by Hsing-Kuo [16]. Study by Hsing-Kuo found that the core values of the WebQuest were to improve students’ capacities for analysis, synthesis, and evaluation, but schools’ mid-term tests could not measure these capacities [16]. According to our observations on teaching, the WebQuest course designed for our research is mainly task-oriented learning, in which students can develop a peer support system in the processes of problem solving or completing learning tasks. The level of cooperation among peers combined with the WebQuest design framework has a great influence on the effectiveness of learning knowledge.

The results of our research have failed to find evidence of the effectiveness of the intervention on improving the attitude towards cyber-bulling. These findings are same as the results of Yen-Yu’s study [19], but different from the results of Wen-Cheh’s study [32]. Yen-Yu’s study found that the course content had less emphasis on developing a concept but had emphases on the learning of knowledge and behavior change [19]. The researcher believes that the small sample size and short experimental sessions are the reasons why our experiment does not have a significant effect. It takes a long time to change the attitude towards cyber-bulling; therefore, it is not easy to change students’ attitudes toward cyber-bullying after only a four-week course. Considering the study as a pilot one, further long experimental sessions with large number of students are warranted which may have different results.

The course for the present study can immediately and effectively reduce the cyber-bullying intention and has after learning retention effects. These results are in line of Kuo-Chin’s study [33]. This is because the course content makes students be aware of the formal rules of the internet usage, enhances the self-protection prevention measures from an internet security viewpoint, and finally guides students to learn responsibilities and justice through the internet law. These have a great influence on the cyber-bulling intention.

Experience of the current study indicates that the teaching methods of the WebQuest course can be vivid and vigorous. The course content can primarily be on a daily-life basis. It is better to give a number of examples to increase students’ awareness and sympathy. Other teachers in the teaching team may be invited to join a discussion and work together on the resources provided by the resource web pages. Students of similar age to the subjects of the research can be invited to provide their views of WebQuest web pages. Other teachers in the team can be invited to come to the classroom to help the researcher keep a record of observations on the classroom. In addition to keeping a record of what happens in the classroom, peer teachers can also give objective advices about the teaching and can support and encourage one another. Information literacy and internet ethics can be added to the course. Other course contents in information field can also be explored. It is warranted that for the related research in the future, the sample sizes of the experimental and the control groups can be increased.

The current study has tested a new intervention with a newly designed scale. The study is a pilot designed as a preliminary test of the measure and the intervention. Based on pilot study purposes and results following conclusions can be drawn. The cyber-bulling prevention WebQuest course can immediately enhance the knowledge of cyber-bulling and has after learning retention effects. The cyber-bulling prevention WebQuest course does not have significant effects upon improving the attitude towards cyber-bulling. The cyber-bulling prevention WebQuest course can immediately and effectively reduce the cyber-bulling intention and has after learning retention effects. However, we need a large-scale study before it’s universal implementation.
